# High Diagnostic Accuracy of Thyroid-Stimulating Hormone (TSH) Receptor Antibodies in Distinguishing Graves’ Disease and Subacute Thyrotoxicosis in the Indian Population

**DOI:** 10.7759/cureus.54303

**Published:** 2024-02-16

**Authors:** Lakshmi T Naga Nitin, Shilpa Lakkundi, Sagar Reddy S L, Dhananjaya M Shanthaiah, Sumanas G Datta, Umalakhmi Annavarapu, Vijaya Sarathi

**Affiliations:** 1 Department of Endocrinology, Vydehi Institute of Medical Sciences and Research Centre, Bengaluru, IND; 2 Department of Pathology, Vydehi Institute of Medical Sciences and Research Centre, Bengaluru, IND; 3 Department of Biochemistry, Vydehi Institute of Medical Sciences and Research Centre, Bengaluru, IND

**Keywords:** 99mtc thyroid scintigraphy, tt3/tt4 ratio, tsh receptor antibodies, subacute thyrotoxicosis, graves’ disease

## Abstract

Background and objective: Thyrotoxicosis is a common clinical condition encountered in endocrine practice. Graves’ disease and subacute thyroiditis are the two common causes of thyrotoxicosis and often have overlapping clinical and biochemical features. 99mTc thyroid scintigraphy is the most commonly used confirmatory test to differentiate the two conditions but is not available in the majority of the second-tier cities of India. However, obtaining thyroid stimulating hormone (TSH) receptor antibodies (TSHrAb), another accurate test to differentiate the two conditions, in second-tier cities by outsourcing to labs in major cities is a feasible option nowadays. However, the data on the performance of TSHrAb to differentiate the two conditions in Indian patients is limited. Hence, we have evaluated the diagnostic accuracy of TSHrAb in the Indian population to differentiate Graves’ disease and subacute thyroiditis.

Materials and methods: This prospective study was conducted on 115 consecutive newly diagnosed thyrotoxicosis patients presenting to the Department of Endocrinology at a tertiary health care centre in India. Clinical parameters like throat pain, duration of symptoms, and grade of goitre were noted. Measurement of total tri-iodothyronine (TT3), total thyroxine (TT4), TSH, TSHrAb, and 99mTc thyroid scintigraphy were performed in all participants. All participants were followed up at least for six months after the recruitment. Increased tracer uptake (>4%) and/or increased thyroid to parotid trace uptake ratio (>2.5) were used to diagnose Graves’ disease.

Results: Eighty-one and 34 patients were diagnosed with Graves’ disease and subacute thyroiditis, respectively. TT3/TT4 ratio had low diagnostic accuracy (area under the curve (AUC): 0.6, best cut-off: 15.6, sensitivity: 53.1%, specificity: 79.4%). TSHrAb had the best AUC (0.9) to distinguish Graves’ disease from subacute thyroiditis (cut-off: 2.0 IU/L, sensitivity: 97.5%, specificity: 100%). In contrast, the kit manufacturer’s reference range (1.75 IU/L) was slightly more sensitive (98.8%), but less specific (94%).

Conclusion: The TT3/TT4 ratio is not a good test to differentiate Graves’ disease and subacute thyroiditis. TSHrAb is accurate in distinguishing Graves’ disease from subacute thyroiditis and a level of 2.0 may be a more accurate cut-off to differentiate the two conditions in the Indian population.

## Introduction

Thyrotoxicosis is a common clinical condition encountered in endocrine practice. Graves’ disease and subacute thyroiditis are the two common causes of thyrotoxicosis and often have overlapping clinical and biochemical features [1].

Historically, ultrasound of the thyroid gland was used to distinguish between Graves' disease and subacute thyroiditis before the advent of current diagnostics. However, it has poor sensitivity and specificity, compared to either thyroid stimulating hormone (TSH) receptor antibody (TSHrAb) or 99mTc-thyroid scintigraphy [2-4] and it can be operator-dependent. Newer diagnostic methods such as 99mTc-thyroid scintigraphy and TSHrAb are the current investigations of choice for differentiating Graves’ disease from subacute thyroiditis.

99mTc-thyroid scintigraphy is the most commonly used confirmatory test to differentiate the two conditions with very high sensitivity (96.6%) and specificity (97.1%) but is not available in the majority of the second-tier cities of India and has certain contraindications [5]. However, the TSHrAb test can be done even in second-tier cities by outsourcing to laboratories in the major cities. The underutilization of TSHrAb in India has also been emphasized recently [6]. 

TSHrAb immunoassays have evolved to become valuable tools in diagnosing and managing Graves' disease and related conditions, with third-generation assays offering high sensitivity and specificity. However, unlike the more complex and resource-intensive bioassays, the commonly used immunoassays that measure TSHrAb can't differentiate between stimulating and blocking antibodies. In a meta-analysis of 21 studies, the pooled sensitivity and specificity of TSHrAb measured with competitive-binding immunoassays were 98% and 99%, respectively [7].

However, data on the performance of TSHrAb to differentiate the two conditions in Indian patients is limited [8]. In contrast to the earlier mentioned meta-analysis [7], a previous study from Kerala noted low specificity (63%) of the manufacturer cut-off (1.75 IU/ml) and suggested a higher cut-off (3.3 IU/ml) as the best cut-off in their cohort [8]. This suggests a need for more studies to evaluate the performance of TSHrAb in the ethnically diverse Indian population. Hence, we evaluated the diagnostic accuracy of TSHrAb in the Indian population to differentiate Graves’ disease from subacute thyroiditis.

## Materials and methods

This was a prospective study conducted at Vydehi Institute of Medical Sciences and Research Centre, a tertiary care center located in Bengaluru, India. The study was approved by the Vydehi Institutional Ethics Committee(approval number: VIEC/2022/APP/008. Adult de-novo detected thyrotoxicosis (low serum TSH with high/normal serum total thyroxine (TT4)) patients who consulted the Department of Endocrinology from July 2021 to December 2022 were included in the study. A detailed history was taken and a clinical examination was performed to look for goiter and signs of thyrotoxicosis and Graves’ disease-associated features. All participants underwent measurement of serum total tri-iodothyronine (TT3), TT4, and TSH and 99mTc-pertechnetate thyroid scintigraphy. Participants who had other causes of thyrotoxicosis (e.g., toxic multinodular goiter, toxic adenoma, amiodarone-induced thyrotoxicosis, and/or exogenous thyroxine intake), acute or chronic medical illnesses, pregnant and lactating women, and women on combined contraceptive pills were excluded. 

Blood samples were collected in a fasting state. Serum TT3, TT4, and TSH were measured by using the chemiluminescent immunoassay method on the Access 2 Immunoassay System (Beckman Coulter, Inc., Brea, California, United States). Measurement of TSHrAb was done using the iFlash 1200 immunoassay Analyzer (Shenzhen YHLO Biotech Co, Shenzhen, China) by electrochemiluminescent immunoassay (ECLIA). The reference values were 0.4-4.2 µIU/ml for TSH, 0.7-2.04 ng/ml for TT3, 5.5-11 µg/dl for TT4, and <1.75 IU/L for TSHrAb. 99mTc pertechnetate thyroid scintigraphy was done using a gamma camera (Infinia; GE Healthcare, General Electric Company, Boston, Massachusetts, United States) in all participants. Increased tracer uptake (>4%) and/or increased thyroid to parotid tracer uptake ratio (>2.5) were used to diagnose Graves’ disease at presentation. Patients with the diagnosis of Graves’ disease were initiated on oral carbimazole, the dose of which was based on the discretion of the treating endocrinologist whereas patients with the diagnosis of subacute thyroiditis were observed without antithyroid drugs. Oral beta-blockers were prescribed if the resting pulse rate was ≥ 90/min. Follow-up thyroid function tests were done once every two months, and all the patients were followed for at least six months.

Collected data was tabulated in MS Excel (Microsoft Corporation, Redmond, Washington, United States) and analyzed using IBM SPSS Statistics for Windows, Version 21.0 (Released 2012; IBM Corp., Armonk, New York, United States). Continuous variables were presented as Mean±SD and categorical variables were presented as frequency and percentage. The chi-square test was used to compare categorical variables and the independent t-test compares parametric continuous variables. Correlation between continuous variables was done using Pearson’s correlation coefficient. Receiver operating characteristic curve (ROC) analysis was done to area under the curve (AUC) for TSHrAb, and TT3/TT4 ratio in differentiating Graves’ disease from subacute thyroiditis. P-value <0.05 was considered as statistically significant.

## Results

Eighty-one and 34 patients were diagnosed with Graves’ disease and subacute thyroiditis, respectively. The mean age of our patients was 34.3±6.9 years, and the majority were women (73%). Age and gender distribution were comparable between Graves’ disease and subacute thyroiditis whereas duration of thyrotoxic symptoms was higher in Graves’ disease (Table 1). Goiter was significantly more frequent in Graves’ disease than subacute thyroiditis (98.5% vs. 70.5%) with the exclusive occurrence of Grade 2 goiter in Graves’ disease (64%). Serum TT3, TT4, and TT3/TT4 ratios were significantly higher whereas serum TSH tended to be lower in patients with Graves’ disease than those with subacute thyroiditis (Table 1). The percentage 99mTc pertechnetate uptake (12.79±9.04% vs. 0.41±0.19%) and TSHrAb (15.04±11.4 vs. 0.89±0.4 IU/L, p<0.0001) were significantly higher in patients with Graves’ disease than those with subacute thyroiditis (Table 1).

**Table 1:** Comparison of demographic, clinical, laboratory, and scintigraphic parameters between patients with Graves’ disease and subacute thyroiditis TT3: total triiodothyronine, TT4: total thyroxine, TSHrAb: thyroid stimulating hormone receptor antibody; TAO: thyroid associated orbitopathy; TSH: thyroid stimulating hormone The categorical data are represented as absolute numbers (n) or percentages (%) and continuous variables are represented as mean ± SD. p-value < 0.05 is considered statistically significant.

		Graves’ disease (n=81)	Subacute thyroiditis (n=34)	p-value
Age (years), mean±SD		34.7±7.2	33.3± 6.1	0.33
Females, n (%)		62 (76.5%)	22 (64.5%)	0.192
Goiter, n (%)	Grade 0	1 (0.01%)	10 (29.5%)	<0.0001
	Grade 1	28 (34.5%)	24 (70.5%)	
	Grade 2	52 (64%)	0	
TAO, n		20	0	NA
Duration of symptoms (months), mean±SD		3.36± 1.51	1.69±0.88	<0.0001
TT3 (ng/ml), mean±SD		3.5±1.5	2.2±1	<0.0001
TT4 (µg/dl), mean±SD		21.1±6.1	15.9±3.7	<0.0001
TT3/TT4 (ng/µg), mean±SD		16.6±5.3	14.1±5.2	0.023
TSH (µIU/ml), mean±SD		0.009±0.01	0.01±0.03	0.051
99m-Tc uptake (%), mean±SD		12.79±9.04	0.41±0.19	<0.0001
TSHrAb (IU/L), mean±SD		15.04±11.4	0.89±0.4	<0.0001

In ROC analysis, duration of symptoms, TT3, TT4, TT4/TT3 ratio, and TSHrAb significantly differentiated Graves’ disease from subacute thyroiditis (Table 2).

**Table 2:** Diagnostic power of clinical and laboratory parameters to differentiate Graves’ disease and subacute thyroiditis on ROC curve TT3: total triiodothyronine, TT4: total thyroxine, TSHRAb: thyroid stimulating hormone receptor antibody, ROC: receiver operating characteristic; AUC: area under the curve p-value of < 0.05 was considered statistically significant.

	AUC	p-value	Best cut-off	Sensitivity	Specificity
Duration of symptoms	0.82	<0.001	2.25 months	70.4%	76.5%
TT3	0.75	<0.001	2.6 ng/ml	70.4%	85.3%
TT4	0.75	<0.001	17.7 µg/dl	69.1%	76.5%
TT3/TT4	0.66	0.006	15.6 ng/µg	53.1%	79.4%
TSHrAb	0.99	0.0001	2 IU/L	97.5%	100%

**Figure 1:**
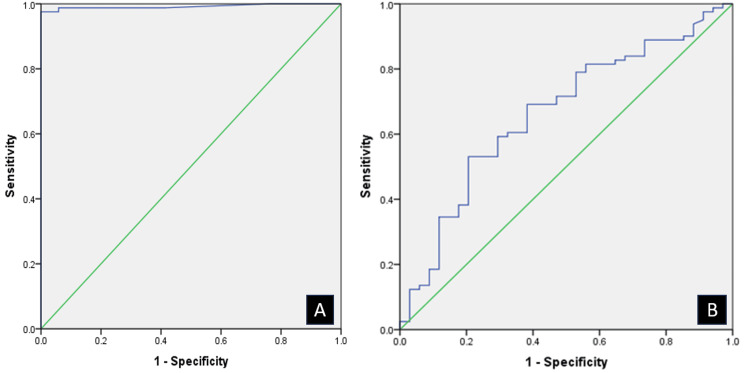
Receiver operating characteristic curve to differentiate Graves’ disease from subacute thyroiditis using TSHrAb (A) and TT3/TT4 ratio (B) TT3: total triiodothyronine, TT4: total thyroxine, TSHRAb: thyroid stimulating hormone receptor antibody

Among these predictors, TSHrAb had the best AUC (0.9) (Figure 1A) to distinguish Graves’ disease from subacute thyroiditis whereas TT3/TT4 ratio had the lowest AUC (0.66) (Figure 1B). The best TSHrAb cut-off obtained was 2.0 IU/L (sensitivity: 97.5%, specificity: 100%). In contrast, the reference range (1.75 IU/L) of the kit manufacturer (Elecsys® Anti-TSHR; Roche Diagnostics Corporation, Indianapolis, Indiana, United States) was slightly more sensitive (98.8%), but less specific (94%). A sensitivity of 100% to diagnose Graves’ disease was obtained at 1.4 IU/L.

## Discussion

In the present study, we report high diagnostic accuracy of TSHrAb to differentiate Graves’ disease and subacute thyrotoxicosis, the two common causes of thyrotoxicosis. The study reports the highest diagnostic accuracy at a cut-off of 2 IU/L which also provides 100% specificity.

The current study included a total of 115 patients with thyrotoxicosis, out of which 81 (70.4%) were diagnosed with Graves’ disease whereas the rest (n=34) were diagnosed with subacute thyroiditis. In most of the centers, Graves’ disease is the most common cause of thyrotoxicosis [9,10]; however, the prevalence of subacute thyroiditis varies markedly. In a study from Kashmir, India, from 1900 to 1997, only 1.5% of the thyrotoxicosis patients were represented by subacute thyroiditis [10]. A systematic review and meta-analysis from Africa reported subacute thyroiditis in only 1% of thyrotoxicosis patients. However, recent studies utilizing nuclear scintigraphy for the evaluation of thyrotoxicosis report subacute thyroiditis in a larger proportion (16-19%) [11]. A recent study has reported a large amount of thyroiditis in Bengaluru, India, based on a nuclear medicine centers-based study, although the proportion of thyroiditis among thyrotoxicosis was not reported, which may indicate a higher prevalence of subacute thyroiditis in the city [12]. The coronavirus disease 2019 (COVID-19) epidemic in 2020-2021 and vaccination for COVID-19 might have also contributed to a slightly higher proportion of subacute thyroiditis in the current study [13,14].

The mean age of Graves’ disease patients was 34.7± 7.2 years and it was 33.3±6.1 years in subacute thyroiditis patients with no significant difference between the two groups (p=0.339). The average age at presentation of Graves’ disease in Indian studies varies from 32 to 47 years [15], whereas it was 34.7± 7.2 years in our study. There was no significant age difference between Graves’ disease and subacute thyroiditis which was also noted in another large study from South India [15]. A female preponderance was noted in both Graves’ disease (76.5%) and subacute thyroiditis (64.5%), which has been well described in the previous Indian studies [15]. Estrogen and partial inactivation of the X chromosome explain female preponderance in both these conditions [16]. The duration of symptoms was longer in patients with Graves’ disease than those with subacute thyroiditis (3.36±1.51 months vs. 1.69±0.88 months, p=<0.0001) and was a useful test to differentiate the two conditions with a relatively better AUC than thyroid function parameters. However, a duration of 2.25 months had the best diagnostic accuracy which is slightly shorter than the conventional cut-off of three months [17].

Total T3/T4 ratio was significantly higher in subjects with Graves’ disease than those with subacute thyroiditis (16.6±5.3 vs 14.1±5.2, p=0.06) as were mean total T3 (3.5 ± 1.5 vs 2.2 ± 1, p=<0.0001 and total T4 levels (21.1 ± 6.1 vs 15.9 ± 3.7, p=<0.0001). Significantly higher thyroid hormone levels and a lower TSH have been reported in patients with Graves’ disease than those with subacute thyroiditis in a previous large study from South India [15]. A more severe disease in Graves’ disease results from increased production of thyroid hormones rather than increased release of the stored preformed hormones, as noted in subacute thyroiditis. A higher total T3/T4 ratio in Graves’ disease is due to increased intrathyroidal deiodinase 1 activity [18]. Notably, none of the thyroid function-related parameters had good diagnostic accuracy. 

We assessed the utility of TSHrAb to differentiate Graves’ disease from subacute thyroiditis in subjects with suppressed TSH. TSHrAb performed with a third-generation ECLIA had a high sensitivity of 98.8% but a relatively lower specificity of 94% when the manufacturer-recommended cut-off of 1.75 IU/L was used [9]. Notably, a recent study from Kerala has demonstrated poor specificity (63%) of the assay at the manufacturer-provided cut-off of 1.75 IU/L [8]. The study recommended 3.37 IU/L as the best cut-off which had 91% and 90% sensitivity and specificity, respectively. We also found a cut-off (2 IU/L) that is slightly higher than the manufacturer-provided cut-off, though with high sensitivity (97.55) and specificity (100%) for diagnosing Graves’ disease. Several studies have demonstrated suboptimal diagnostic accuracy [19-21]. As 100% sensitivity was obtained at 1.4 IU/L, we suggest 99m-Tc thyroid scintigraphy for thyrotoxicosis patients with TSHrAb between 1.4 and 2.0 IU/L is prudent. 

A few studies have compared the performance of different assays for the measurement of TSHrAb. In a recent study comparing two third-generation immunoassays for the measurement of TSHrAb, a high agreement between the two methods was obtained (kappa; 0.82) [22]. Another recent study demonstrated slightly better diagnostic performance of third-generation enzyme-linked immunosorbent assay (ELISA) methods than second-generation ones [23]. Recent studies have also demonstrated the utility of a few newer methods for the measurement of TSHrAb such as one-step third-generation radioimmunoassay and dot-blot method are useful, albeit with modestly lower diagnostic accuracy of the latter method [24,25]. Thyroid stimulating antibody (TSI), the best method for assessment of autoimmunity in Graves’ disease, may have better diagnostic performance in diagnosing Graves' disease, and offer a better prediction of thyroid-associated orbitopathy and response to antithyroid therapy [26-28]; however, the reports are variable and advantages in clinical practice are minimal [29]. Moreover, this assay is not currently available in India. Hence, the measurement of TSHrAb by a third-generation immunoassay is an apt biochemical method to diagnose Graves’s disease in India.

Some limitations of our study were its monocentric nature and lack of comparison with other immunoassays. However, this was a prospective study with validation of the diagnosis not only against 99m-Tc thyroid scintigraphy but also against follow-up for at least six months.

## Conclusions

TSHrAb measured by a third-generation assay (ECLIA) was accurate in distinguishing Graves’ disease from subacute thyroiditis and a level of 2.0 IU/L may be a more accurate cut-off to differentiate the two conditions in the Indian population than the manufacturer-provided cut-off of 1.75 IU/L. We suggest obtaining 99m-Tc pertechnetate scintigraphy in thyrotoxicosis patients with TSHrAb levels between 1.4 and 2.0 IU/ml. 
